# 

**Published:** 1876-06

**Authors:** 


VOL. VI. JUNE, 1876. NO. 6.

### THE SOUTHERN MEDICAL RECORD:

PUBLISHED MONTHLY AT

### ATLANTA, GEORGIA.

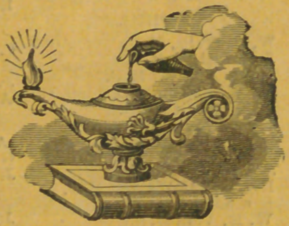

LOCAL EDITORS:

THOS. S. POWELL, M.D.

R. C. WORD, M.D

W. T. GOLDSMITH, M.D.

H. B. LEE, M.D.

ASSOCIATE EDITORS:

T. CURTIS SMITH, M.D.......... .....MIDDLEPORT, OHIO.
SWAN M. BURNETT, M.D .......KNOXVILLE, TENN.
ALBAN S. PAYNE, M.D.......... .....MARKHAM'S, VA.
A. R. KILPATRICK, M.D..........NAVASOTA, TEXAS.
A. A. LYON, M.D..... ..................SHREVEPORT, LA.
G. A. BAXTER, M.D ..............CHATTANOOGA, TENN.
J. B. MATTISON, M.D.............BROOKLYN, N. Y.
E. T. EASLY...... .. ............... .....LITTLE ROCK, ARK

*"QUICQUID PRÆCIPIES ESTO BREVIS."*

ATLANTA, GA.:

JAS. P. HARRISON & CO., PRINTERS AND BINDERS.

1876.

